# Abnormal Changes in NKT Cells, the IGF-1 Axis, and Liver Pathology in an Animal Model of ALS

**DOI:** 10.1371/journal.pone.0022374

**Published:** 2011-08-02

**Authors:** Arseny Finkelstein, Gilad Kunis, Akop Seksenyan, Ayal Ronen, Tamara Berkutzki, David Azoulay, Maya Koronyo-Hamaoui, Michal Schwartz

**Affiliations:** 1 Department of Neurobiology, The Weizmann Institute of Science, Rehovot, Israel; 2 Department of Neurosurgery, Cedars-Sinai Medical Center, Los Angeles, California, United States of America; 3 Department of Veterinary Resources, The Weizmann Institute of Science, Rehovot, Israel; Centre de Recherche Public de la Santé (CRP-Santé), Luxembourg

## Abstract

Amyotrophic lateral sclerosis (ALS) is a rapidly progressing fatal neurodegenerative disorder characterized by the selective death of motor neurons (MN) in the spinal cord, and is associated with local neuroinflammation. Circulating CD4^+^ T cells are required for controlling the local detrimental inflammation in neurodegenerative diseases, and for supporting neuronal survival, including that of MN. T-cell deficiency increases neuronal loss, while boosting T cell levels reduces it. Here, we show that in the mutant superoxide dismutase 1 G93A (mSOD1) mouse model of ALS, the levels of natural killer T (NKT) cells increased dramatically, and T-cell distribution was altered both in lymphoid organs and in the spinal cord relative to wild-type mice. The most significant elevation of NKT cells was observed in the liver, concomitant with organ atrophy. Hepatic expression levels of insulin-like growth factor (IGF)-1 decreased, while the expression of IGF binding protein (IGFBP)-1 was augmented by more than 20-fold in mSOD1 mice relative to wild-type animals. Moreover, hepatic lymphocytes of pre-symptomatic mSOD1 mice were found to secrete significantly higher levels of cytokines when stimulated with an NKT ligand, *ex-vivo*. Immunomodulation of NKT cells using an analogue of α-galactosyl ceramide (α-GalCer), in a specific regimen, diminished the number of these cells in the periphery, and induced recruitment of T cells into the affected spinal cord, leading to a modest but significant prolongation of life span of mSOD1 mice. These results identify NKT cells as potential players in ALS, and the liver as an additional site of major pathology in this disease, thereby emphasizing that ALS is not only a non-cell autonomous, but a non-tissue autonomous disease, as well. Moreover, the results suggest potential new therapeutic targets such as the liver for immunomodulatory intervention for modifying the disease, in addition to MN-based neuroprotection and systemic treatments aimed at reducing oxidative stress.

## Introduction

Amyotrophic lateral sclerosis (ALS) also known as Lou Gehrig's disease is a rapidly progressing fatal neurodegenerative disorder, with death occurring typically within 2–5 years after the appearance of clinical symptoms. While the majority of ALS cases are sporadic, 10 percent of them are inherited, with the most abundant mutation occurring in the superoxide dismutase (SOD)-1 gene [1]. In both its sporadic and genetic manifestations, disease progression has been attributed to a selective death of motor neurons (MN) in the spinal cord, with evidence for neuroinflammation and acquisition of a cytotoxic phenotype by resident microglia [2], [3], [4].

Adaptive immunity is required to control the local detrimental inflammation in neurodegenerative diseases, and is beneficial for neuronal survival [5], [6], [7], [8], [9]. Specifically, it was shown that T cells play a protective role in MN degeneration in the mutant superoxide dismutase 1 G93A (mSOD1) mouse model of ALS [10], [11], [12]. However, during the progression of this disease, a systemic immune pathology involving thymic involution develops, possibly reducing the naturally occurring neuroprotective immune response [10], [13].

Regulatory cells of the immune system include naturally occurring and induced CD4^+^CD25^+^ Treg cells, myeloid suppressor cells, CD8^+^ regulatory cells, and natural killer T (NKT) cells [14], [15], [16], [17]. NKT cells share markers with both Natural Killer (NK) and conventional T cells and are particularly abundant in the liver [18], [19]; the most well studied population is the invariant NKT (iNKT) cell subset, which recognizes glycolipid antigens presented by the non-classical major histocompatibility complex (MHC)-I-like molecule, CD1d [20]. Upon activation by the prototypical antigen, α-galactosyl ceramide (α-GalCer), or its analogue, PBS57, these cells rapidly release large amounts of cytokines [20], [21].

NKT cells may be particularly active in neuroinflammation, since the central nervous system (CNS) is enriched with glycolipids [22]. Furthermore, as the number of NKT cells is reduced in many autoimmune diseases, including multiple sclerosis, these cells were suggested to act as suppressors of autoimmune reactions [15], [23], [24]. However, their role in other neurodegenerative diseases with a local neuroinflammatory response remains unclear.

Here we found that NKT cells are elevated in the lymphoid organs and CNS of mSOD1 mice. The most dramatic elevation was observed in the liver, where NKT cells became the predominant lymphocyte cell population, concomitantly with liver atrophy, changes in IGF-1 axis, and pathological relocation of lipids across the tissue. Administration of PBS57, a glycolipidic ligand of NKT cells, in a specific regimen, delayed the onset of the disease and prolonged the survival of mSOD1 mice. The beneficial outcome of the treatment was correlated with down-regulation of peripheral NKT cells and recruitment of T cells into the affected spinal cord.

## Materials and Methods

### Animals

Wild-type (WT) and mSOD1^G93A^ mice on C57Bl or C57Bl/SJL background were supplied by Harlan Biotech (Jerusalem, Israel), the Animal Breeding Center of The Weizmann Institute of Science, and the Jackson Laboratory.

### Ethics statement

The animals were treated and handled according to the guidelines of the Weizmann Institute's Animal Care and Use Committee. The study was approved by the Animal Care and Use Committee of the Weizmann Institute, permit number: 03090608-1.

### Administration of PBS57

A stock solution of PBS57 containing 2 mg/ml PBS57 in dimethyl sulfoxide (DMSO) was dissolved in phosphate-buffered saline (PBS) and injected i.p. (2 µg per mouse) in a final volume of 150 µl. Mice on a C57Bl/SJL background received a single injection of PBS57 at day 60, and then four additional weekly injections starting from the age of 95 days. Mice on a C57Bl background received one injection at day 70±2, and then two weekly injections starting from the age of 105±2 days.

PBS57 was kindly provided by Dr. Paul Savage (Department of Chemistry and Biochemistry, Brigham Young University, Provo, Utah).

### Rota-rod

Prior to the development of symptoms, mice were trained to stay on the Rota-rod (accelerating Rota-rod for mice 7650, Jones and Roberts) with increasing velocity for 3 minutes. Starting from 95 days of age, their performance was assessed twice per week with three daily sessions, by taking the best of the three sessions. Onset age was defined as the age of the animal at the day when it failed to complete the 3 minute run during all three sessions.

### Flow cytometry

Mice at different stages of disease were perfused from the left ventricle with PBS, and their spleens, livers, and spinal cords were collected. The spleens were mashed, and the red blood cells were lysed using ACK lysis buffer (Biosource, Rockville, MD.). Spinal cords and livers were homogenized manually or using a software controlled sealed homogenization system (Dispomix; http://www.biocellisolation.com) followed by separation on a 40% Percoll (GE Healthcare, Sweden) gradient to eliminate residual fat tissue. Liver cell suspension was lysed with ACK lysis buffer following the gradient separation. For florescence-activated cell sorting (FACS) analysis, the following fluorochrome-labeled mAbs were used according to the manufacturers' protocols: FITC conjugated anti-NK1.1, TCRβ, Ly6C, LFA-1, and CD69; PE conjugated anti-CD4, NK1.1, CD1d, and DX5; APC conjugated anti-TCRβ, CD11b, and FOXP3 (BD Pharmingen and eBioscience). APC-conjugated α-GalCer-loaded CD1d tetramers (ProImmune) were used according to the manufacturer's protocol. Stained cells were acquired using FACScan or Cyan flow cytometer and analyzed using CellQuest (BD Biosciences), FlowJo (Tree Star), and Summit (Dako cytometry) software.

### Histology

After perfusion of the mice with PBS, the livers and spinal cords were excised and fixed in 2.5% paraformaldehyde or Bouin, for 48 hours and then placed in 70% EtOH. The tissue was dehydrated sequentially in EtOH:xylene:paraffin over a gradient of 70∶95∶100% and then embedded in paraffin. Sections (6 µm thick) were cut and stained using Nissl, periodic acid-Schiff (PAS) reagent, hematoxylin and eosin.

### Immunohistochemistry

Paraffin sections were prepared and stained, as described above. The slides were then treated with 10 mM sodium citrate (pH 6.0) by heating in the microwave to the boiling point, and then for a further 10 min at 20% microwave power. Blocking was performed with 20% normal horse serum with 0.05% saponin for 60 min. Primary antibody mixture containing 2% normal horse serum and 0.05% saponin was applied for 24 h at 4°C in a humidified chamber. For labeling of myeloid cells, FITC-conjugated *Bandeiraea simplicifolia* isolectin B4 (IB-4, 1∶50; Sigma-Aldrich) and rat anti-CD11b Abs (1∶50, BD Pharmingen, Franklin Lakes, NJ) were used. For labeling of astrocytes, rabbit anti-glial fibrillary acidic protein (anti-GFAP) Abs were used (1∶200; DAKO, Glostrup, Denmark). Secondary antibodies included Cy-2-conjugated donkey anti-mouse, Cy-3-conjugated donkey anti-mouse, Cy-3- donkey anti-rat, and Cy-3-conjugated donkey anti rabbit (1∶200; Jackson ImmunoResearch, West Grove, PA). For nuclear staining, Hoechst 33342 fluorochrome was used (Molecular Probes Invitrogen). The stained sections were mounted with GVA mounting solution (Invitrogen).

### Quantitative real time polymerase chain reaction (Q-PCR)

Total cellular RNA purification and cDNA synthesis was performed as described previously [5]. Q-PCR reactions were performed with a high-speed thermal cycler (LightCycler; Roche Diagnostics Corp.), and the product was detected by FastStart Master SYBR Green I (Roche Molecular Biochemicals) according to the manufacturer's instructions. The amplification cycle was 95°C for 10 seconds, 60°C for 5 seconds, and 72°C for 10 seconds. Melting curve analysis confirmed that only a single product was amplified.

The following primers were used:

TNF-α forward 5.-ACAAGGCTGCCCCGACTAT-3.; reverse 5.-CTCCTGGTATGAAGTGGCAAATC-3.

IGF-1 forward 5.-CCGGACCAGAGACCCTTTG-3.; reverse 5.-CCTGTGGGCTTGTTGAAGTAAAA-3.

IGF-1R forward 5.-ATCCTGTGTTCTTCTATGTCC-3.; reverse 5.-CCAACCTGCTGTTATTTCTC-3.

IGFBP-1 forward 5.-CCCAACAAAAGCAGGAG-3.; reverse 5.-TGTCTCACACTGTTTGCTG-3.

IGFBP-3 forward 5.-GAGACAGAATACGGTCCC-3.; reverse 5.-CCTTCTTGTCACAGTTTGG-3.

### Measurement of cytokine production by hepatic lymphocytes

Hepatic lymphocytes were prepared as described above, and seeded (10^5^ cells/well) in triplicates in 96 well plates in a final volume of 200 µl RPMI-1640 medium containing 2.5% fetal calf serum, 2 mM L-glutamine, 1 mM sodium pyruvate, 50 µM β-mercaptoethanol, 100 U/ml penicillin, and 100 µg/ml streptomycin, and either supplemented with 100 ng/ml of PBS57 or left untreated. The cells were incubated at 37°C/5% CO_2_ for 2 d. Cytokine production was determined by enzyme-linked ImmunoSorbent assay (ELISA) of the cell medium, using a kit (eBioscience) according to the manufacturer's protocol.

### Protein extraction

Spleen samples were weighed and then homogenized in cold extraction buffer (Tris-buffered saline, pH 8.0, with 1% NP-40, 10% glycerol, 5 mM sodium metavanadate, 10 mM PMSF, 100 µg/ml aprotinin and 10 µg/ml leupeptin). Homogenates were then centrifuged at 7000 g for 10 min, and supernatants were assayed by ELISA.

### Statistical analysis

Student's t-test and analysis of variance (ANOVA) were considered significant at p<0.05. Kaplan-Meier survival curves were analyzed by Logrank test to generate an χ^2^ value for significance. Statistical calculations were performed using standard functions of Microsoft Excel, JMP, and Stat View software.

## Results

### Accumulation of NKT cells in the spinal cord, spleen and liver of mSOD1 mice

**Figure 1:**
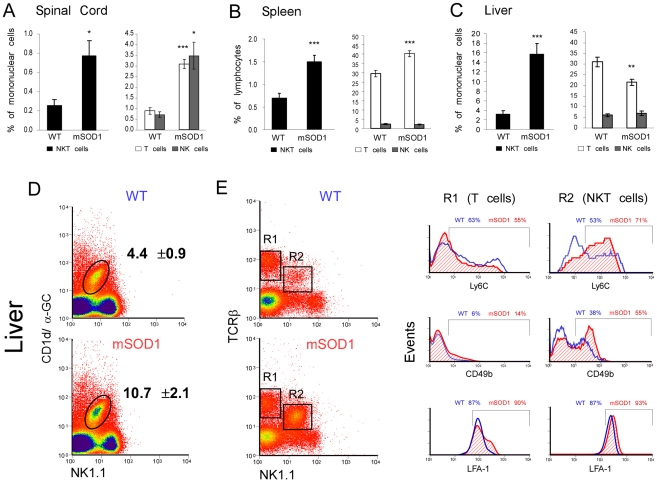
Accumulation of NKT cells in mSOD1 mice. FACS analyses (**A–C**) of the proportion of NKT, T, and NK cells in the spinal cord (n = 4–7 per group), spleen, and liver (n = 10–14 per group) in WT or mSOD1 mice on C57Bl/SJL background at the end-stage of the disease (day 135). (**D**) The proportion of hepatic iNKT cells (n = 4 per group), analyzed by expression of TCRβ and binding of α-GalCer-loaded CD1d tetramers (CD1d/α-GC). (**E**) Hepatic lymphocytes gated on T (R1) or NKT (R2) cells were analyzed for markers of activation state (CD49b) and migratory ability (Ly6c, LFA-1). Data are expressed as mean±SEM; *p<0.05, **p<0.01, ***p<0.001 versus WT mice by Student's t-test.

**Figure 2:**
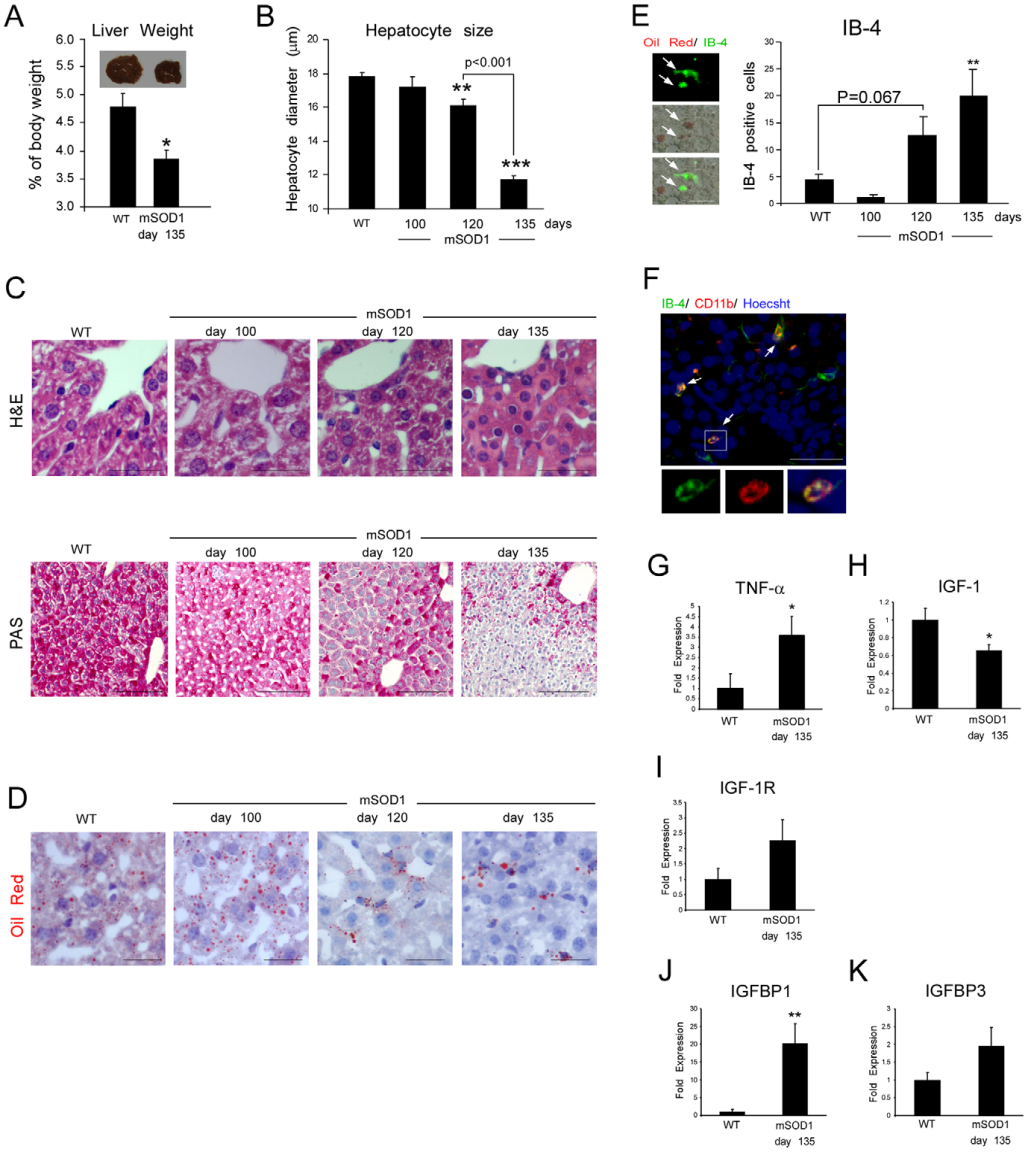
Liver pathology is associated with inflammation and changes in IGF-1 axis in mSOD1 mice. (**A**) Liver weight relative to body mass of WT and mSOD1 mice on C57Bl/SJL background, and representative pictures of the tissue. (**B**) Quantitative analysis of hepatocyte diameter using Hematoxylin & Eosin (H&E) stained liver. (**C**) Representative micrographs of H&E and PAS stained livers depicting changes in hepatocyte size and reduction in PAS-positive material. Scale bars represent 50 µm and 100 µm, respectively. Note the loss of granular composition in the cytoplasm along the course of disease progression. (**D**) Liver staining with Oil Red demonstrating marked lipid redistribution in the disease. Scale bar is 25 µm. Specifically, lipid droplets accumulated in extracellular spaces, or were partially co-localized (**E**) with IB-4 positive cells (quantified at different stages of the disease; arrows indicate double-labeled cells). Scale bar represents 25 µm. (**F**) Representative micrograph of liver sections stained for IB-4 (green), CD11b (red), and Hoechst staining for nuclear content (blue). Scale bar represents 50 µm. The majority (up to 80%) of IB-4^+^ cells also expressed CD11b; arrows indicate double-labeled cells (split images include high magnification fields of the white boxed area). (**G–K**) Expression of TNF-α, IGF-1, IGF-1R, IGFBP1, and IGFBP3 from liver samples of WT and mSOD1 mice at day 135, as determined by Q-PCR. Data are expressed as mean±SEM (n = 3–6 per group). *p<0.05, **p<0.01, ***p<0.001 versus WT mice by one-way ANOVA followed by Student's t-test *post-hoc* analysis.

The local inflammation in ALS, together with the compromised protective immune response [10], [13], encouraged us to search for novel immunoregulatory targets in this disease. The reported reduction of NKT cells in inflammatory autoimmune diseases [23], [25], [26], on one hand, and the need for autoimmune protective T cells in non-inflammatory neurodegenerative diseases such as ALS [27], on the other hand, prompted us to analyze the fate of these cells in ALS. We first examined whether there is any homing of NKT cells to the affected spinal cord of C57Bl/SJL mSOD1 mice, and found that their proportion increased significantly at the clinical end-stage of the disease (**Fig. 1A**). In the spleen, despite a reduction in its size, the abundance of NKT was also found to be significantly in mSOD1 mice relative to WT (**Fig. 1B**), possibly resulting from lymphopenic-driven proliferation [10]. We found the most prominent increase in the proportion of NKT cells in the liver, which also decreased in size as the disease progressed (see **Fig. 2** and the section below) (**Fig. 1C**). In fact, the proportions of NKT cells in the liver increased by 4-fold at the end-stage of the disease. NKT-cell levels differ in mice of different genetic backgrounds [28]. We therefore also measured the fate of NKT cells in mSOD1 mice on a C57Bl background, and found similar dynamics to the C57Bl/SJL background, with the hepatic NKT cells becoming the predominant lymphocyte population with the disease progression (**Fig. S1**).

Since spleen [10], thymus [13], and liver (**Fig. 2A**) all shrink in size during disease progression, the change in lymphocyte proportions in these organs does not necessarily reflect a change in their absolute number. In order to investigate if the increase in NKT cell population is accompanied by changes in the abundance of other immune cells that share common markers with NKT cells, we also determined the proportions of T cells and NK cells in different organs of mSOD1 mice at the end-stage of the disease. T cells were significantly elevated in the spinal cord and spleen of mSOD1 mice relative to WT, although to a lesser extent when compared to NKT cells (**Fig. 1A–B**). No significant change in abundance of Treg cells was observed in the spleen (data not shown). In contrast, the change in the proportion of T cells in the liver did not mirror the corresponding change in NKT cells, as T cell abundance was significantly lower in mSOD1 liver relative to WT (**Fig. 1C**). NK cells were found to be elevated significantly only in the spinal cord, but remained unchanged in the spleen and the liver (**Fig. 1A–C**). Thus, the changes in the proportion of NKT cells across different organs, do not merely reflect alterations occurring in the populations of T or NK cells.

To further characterize the population of NKT cells in the periphery, we analyzed the phenotype of these cells in the lymphoid organs of mSOD1 mice. The proportion of the CD1d-reactive iNKT cell subpopulation was increased in the liver, spleen, and thymus (**Fig. 1D** **and Fig. S2**). Furthermore, NKT cells from mSOD1 mice expressed higher levels of the activation marker, CD69, in the spleen and thymus (**Fig. S2**). Interestingly, the migratory marker Ly6C and the integrin marker, CD49b, associated with cell adhesion, were up-regulated on NKT but not on conventional T cells in the liver, suggesting that hepatic NKT cells acquire an activated state in mSOD1 mice (**Fig. 1E**). The migratory protein, lymphocyte function associated antigen (LFA)-1, remained constant on lymphocytes of mSOD1 mice relative to WT, but a higher basal expression level was detected on NKT cells compared to conventional T cells (**Fig. 1E**). Collectively, these results identify specific changes in lymphocyte populations in the liver of mSOD1 mice, and suggest that accumulation of NKT cells in the CNS might stem from their selective recruitment from the periphery.

### Liver pathology, inflammation, and changes in IGF-1 axis in mSOD1 mice

Since the most notable elevation in NKT cells was observed in the liver, we searched for pathological changes in this organ, and found that its absolute weight, as well as its proportion relative to body mass, was significantly reduced in mSOD1 mice (**Fig. 2A**). The liver weight reduction was accompanied by hepatocyte atrophy (**Fig. 2B,C**), and weak staining with periodic acid-Schiff reagent (PAS), possibly indicating a depletion of glycogen storage (**Fig. 2C**). Staining with Oil Red revealed lipid aggregation during the course of disease progression (**Fig. 2D**), partially co-localized with, or surrounded by isolectin (IB)-4 binding cells (**Fig. 2E**). A marked elevation in IB-4 stained cells was evident in the diseased animals (**Fig. 2E**). In order to further characterize the identity of the IB-4^+^ cells found in the liver, we stained them with the myeloid marker CD11b and found that these markers were colocalized (**Fig. 2F**). Since CD1d is involved in glycolipid presentation to NKT cells [18], [20], we looked for expression levels of CD1d on CD11b^+^cells; no further elevation relative to WT was found in the diseased animals (data not shown). It is therefore possible that the pathological conditions that develop in the liver may be associated with an increase in the proportion of antigen presenting cells for the NKT population. In addition, we found in the liver a high expression levels of tumor necrosis factor (TNF)-α (**Fig. 2G**), indicating that the disease manifestation was correlated with a hepatic inflammatory response. Since the liver is the main source of IGF-1, a key MN survival protein in ALS [29], we measured its expression in the liver and found a significant reduction in mSOD1 mice (**Fig. 2H**). We also observed a slight increase in IGF-1 receptor (IGF-1R) in the liver that was not statistically significant (**Fig. 2I**). IGF-1 availability and its biological effects are strongly regulated by IGF-1 binding proteins (IGFBPs), which are presumably synthesized and released into the circulation by the liver [30]. Measurement of IGFBP-1 revealed a dramatic increase (by more than 20 fold) in its expression levels in the liver of mSOD1 mice relative to WT (**Fig. 2J**). Testing IGFBP-3 revealed a slight increase that was not statistically significant (**Fig. 2K**). Taken together, these results suggest that the disease progression in mSOD1 mice is correlated with liver inflammation, atrophy, and a decrease in the production and possible availability of IGF-1.

### NKT cell-based immunomodulation delays the onset of clinical symptoms and prolongs the survival of mSOD1 mice

**Figure 3:**
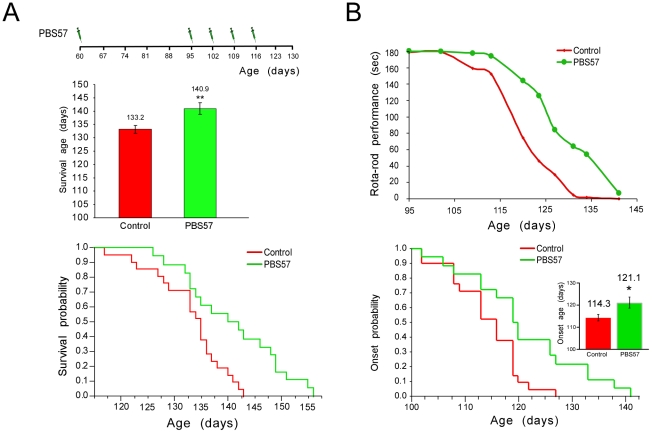
Administration of PBS57 extends survival of mSOD1 mice. (**A**) Repeated administration of PBS57 to mSOD1 female mice on C57Bl/SJL background according to the described regimen resulted in increased survival, as shown by average survival time and corresponding Kaplan-Meier curves for untreated (n = 21) versus PBS57 treated (n = 18) mice (Logrank χ^2^ = 8.9, p<0.005). (**B**) Rota-rod performance of control (n = 21) and PBS57-treated (n = 18) mice (repeated measures ANOVA of the effect of the treatment on Rota-rod performance. Groups: p = 0.069; days: p = 0.0001; groups*days: p = 0.0092). Onset age was defined as the age of the animal at the day it failed to complete a 3 minute run on at least one of three sessions on the Rota-rod. Inset displays the average onset age (Logrank χ^2^ = 6.69, p<0.01). Note a significant delay in clinical disease onset in treated mice. Data are expressed as mean ± SEM. * p<0.05, ** p<0.01 by Student's t-test analysis.

Since we found that NKT cell abundance increased in mSOD1 mice, we envisioned that these cells might be involved in disease progression. One possible mechanism for such an involvement could be via suppression of peripheral immunity needed for fighting off the disease [31]; thus, their manipulation might provide an effective therapeutic approach. We therefore focused on the immunomodulatory ligand, α-GalCer [20], which was shown to induce anergy and hypo-responsiveness of NKT cells following repeated administration [32]. To test the effect of manipulation of NKT cells on disease progression, we subjected mSOD1 mice to treatment with a soluble α-GalCer analog, PBS57 [21]. The animals received a single injection of PBS57 at day 60, and then four additional weekly injections starting from the age of 95 days (according to the regimen described in **Fig. 3A**). A significant prolongation of lifespan (by 7 days) was observed in the treated animals (**Fig. 3A**) and the first appearance of clinical symptoms was also significantly delayed in the PBS57 treated group, by 7 days (**Fig. 3B**), suggesting that manipulating NKT cells attenuated the disease progression at the preclinical stage. The outcome of the treatment was very sensitive to the regimen; no effect on survival or on clinical symptoms (with a non-significant trend for an adverse effect) was observed in animals that received PBS57 weekly starting from day 60 (data not shown). Therefore, in all subsequent experiments we used only the protocol of a first injection on day 60, and weekly injection from day 95 onward. These results suggest that the treatment with PBS57 increases the life span of the animals by delaying the onset of the clinical symptoms of the disease, rather than prolonging the progression phase.

### Treatment with PBS57 delays MN death and reduces astrogliosis in the spinal cord

**Figure 4:**
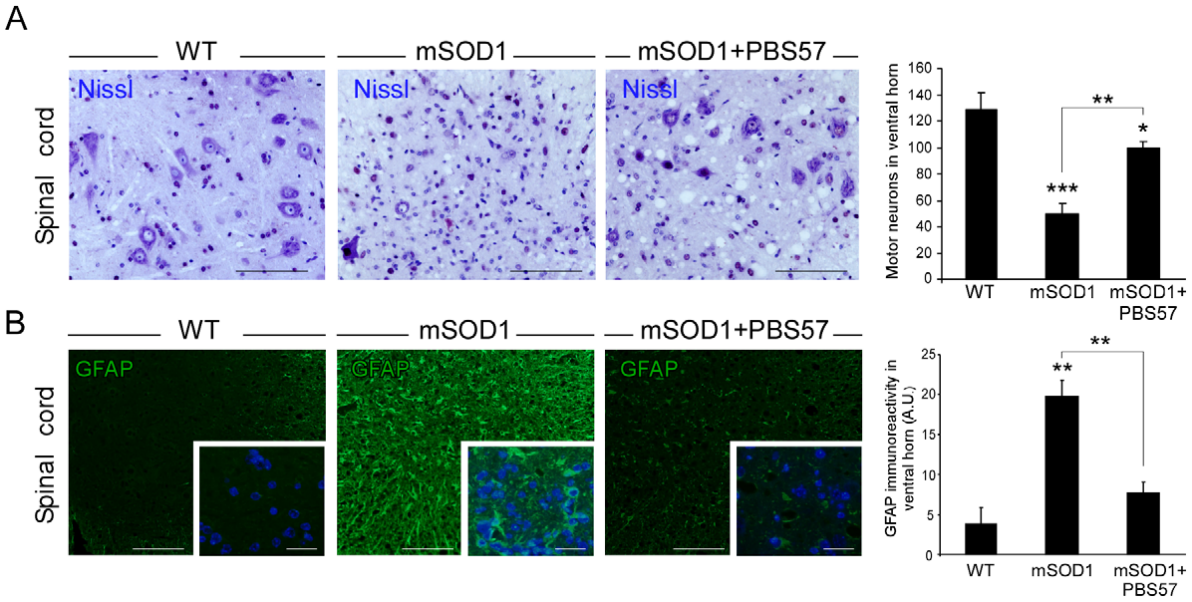
Administration of PBS57 delays MN death in mSOD1 mice. (**A**) Representative micrographs of spinal cord sections at end-stage disease, and quantitative analysis of the number of MNs detected by Nissl staining in WT mice and mSOD1, relative to PBS57-treated littermates. Scale bars are 100 µm. (**B**) Representative micrographs and quantitative analysis of GFAP-reactive astrocytes in the affected spinal cord. Glial activation and tissue disorganization were reduced by PBS57 treatment (insets are high magnification enlargements of white-boxed areas with Hoechst staining for nuclear content in blue). Scale bars in B correspond to 100 µm, insets to 25 µm. Data are expressed as mean±SEM (n = 3-6 per group). *p<0.05, **p<0.01, ***p<0.001 versus WT mice by Student's t-test, or one-way ANOVA followed by Student's t-test *post-hoc* analysis.

We next wished to determine whether immunomodulation of NKT cells could affect the MN pathology observed in the spinal cord. To that end, we counted and compared the number of MNs in lumbar spinal cord sections of untreated mSOD1 mice at age 130–135 days (the average age of death of this group) with that of age-matched PBS7 treated mSOD1 mice. A marked reduction in the number of MNs was observed in mSOD1 untreated mice, while treatment with PBS57 resulted in a significant attenuation of MN loss (**Fig. 4A**). Since glial pathology is one of the hallmarks of ALS [33], [34], [35], we tested whether administration of PBS57 affected astrocyte activation, by staining for glial fibrillary acidic protein (GFAP). GFAP expression was abundant throughout the lumbar part of the spinal cord in mSOD1 mice, while in PBS57-treated animals it was significantly reduced (**Fig. 4B**). In fact, in PBS57-treated animals, GFAP expression was restricted only to the caudal sections, indicating that disease progression in the caudal-rostral direction is attenuated by the treatment.

### Modulation of NKT cells induces a cytokine shift in the liver, and recruits T cells into the spinal cord

**Figure 5:**
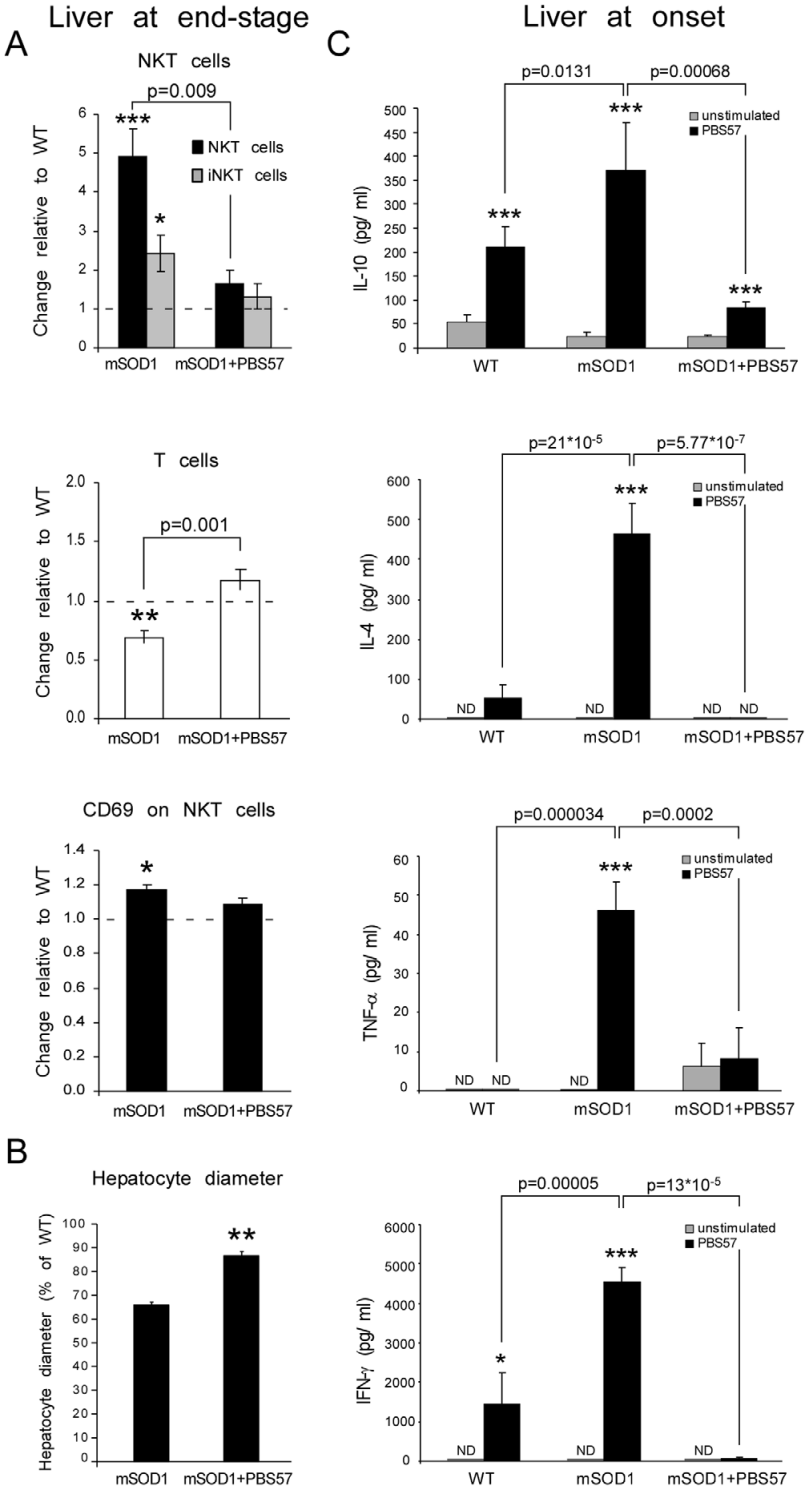
Immunomodulation with PBS57 downregulates NKT cells and increases T cells in the liver. (**A**) NKT, iNKT, and T cells were monitored at the end-stage of the disease in the liver of PBS57-treated or untreated mSOD1 mice on C57Bl/SJL background. The proportion of each cell population and the activation state of NKT cells (defined by CD69 expression) are shown normalized to values obtained for WT controls. (**B**) Diameter of hepatocytes was measured in untreated mSOD1 at the end-stage of the disease and in age-matched PBS57 treated mSOD1 mice, and normalized to levels observed in WT animals. (**C**) Hepatic lymphocytes, harvested from WT or mSOD1 mice 3 days after the 3rd injection of PBS57 (day 105), were tested for cytokine production following *ex-vivo* stimulation. *In-vivo* treatment with PBS57 resulted in abrogation of cytokine production following subsequent *ex-vivo* stimulation, with some retention of IL-10 secretion. Data are expressed as mean±SEM (n = 3–6 per group). *p<0.05, **p<0.01, ***p<0.001 versus WT mice or unstimulated lymphocytes by Student's t-test, or one-way ANOVA followed by Student's t-test *post-hoc* analysis.

To gain insight into the immunological effect of PBS57, the fate of NKT cells following the treatment was assessed. In PBS57-treated mSOD1 mice we found a significant reduction in the proportion of NKT cells in the spleen, thymus, and liver compared to untreated mSOD1 mice at the end-stage of the disease (**Fig. 5A** **and Fig. S3A**). These results are in line with reports that repeated administration of α-GalCer depletes the number of NKT cells and down-regulates their activation state [32], [36]. In addition, we found that the hepatic T-cell population was restored to the levels observed in the WT animals (**Fig. 5A**). PBS57 administration also resulted in a significant reduction in CD69 expression on NKT cells (**Fig. 5A** **and Fig. S3A**), suggesting that the decrease in NKT cells was accompanied by modulation of their activation state, as well. Notably, treatment with PBS57 resulted in attenuation of liver atrophy (**Fig. 5B**), without affecting the expression levels of IGF-1 (data not shown).

Since we observed that PBS57 treatment delayed the onset of clinical symptoms and reversed the accumulation of NKT cells in the late stage of the disease, we further investigated whether any pathological changes could be observed in hepatic lymphocytes before the first appearance of clinical symptoms (around day 105). We did not find any changes in the proportions of NKT cells or T cells in the liver of pre-symptomatic mSOD1 mice relative to WT (data not shown). The above results prompted us to explore whether there were any early pathological changes in the cytokine profile of hepatic lymphocytes in mSOD1 mice, and whether these changes were influenced by therapeutic intervention with PBS57. To that end, hepatic lymphocytes were harvested from WT, mSOD1 control, and mSOD1 PBS57-treated mice, and were tested for cytokine secretion *in-vitro* with and without further *ex-vivo* treatment with PBS57. Importantly, we found that *ex-vivo* activated lymphocytes, harvested from untreated mSOD1 mice, secreted higher levels of all tested cytokines (interleukin (IL)-10, IL-4, TNF-α, and interferon (IFN)-γ) relative to cells harvested from WT mice, with the most pronounced increase in TNF-α (**Fig. 5C**). In contrast, hepatic lymphocytes that were harvested from mSOD1 mice 3-days after the third injection of PBS57 (when the mice were 105 days old), and cultured *ex-vivo* with PBS57 showed a marked reduction in the capacity to secrete these cytokines, with some retention of IL-10 secretion (**Fig. 5C**). Despite the suppression of the capacity of NKT cells to induce cytokine release upon stimulation, the treatment did not result in an overall state of immunosuppression; when we quantified the actual cytokine levels in tissue-extracts, we found a marked increase in the levels of IFN-γ in the total spleen extracts of PBS57-treated mice **(Fig. S3B)**.

**Figure 6:**
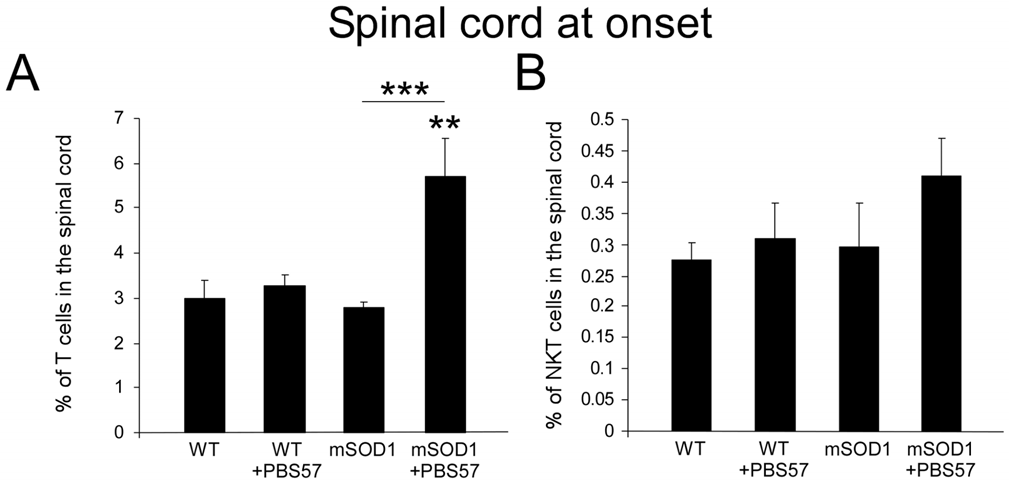
Immunomodulation with PBS57 recruits T cells into the spinal cord of mSOD1 mice. Lymphocytes were harvested from the spinal cord of WT and mSOD1 mice on a C57Bl background 3 days after the 3rd injection of PBS57 (day 115), and were tested for the proportion of T cells (**A**), and NKT cells (**B**) by FACS. A robust increase in the proportion of T cells was observed in the spinal cord of mSOD1 mice that were treated with PBS57. Notably, the treatment did not affect the proportions of lymphocytes in the spinal cord of healthy WT animals. Data are expressed as mean±SEM (n = 3-6 per group). **p<0.01, ***p<0.001 versus WT mice by one-way ANOVA followed by Student's t-test *post-hoc* analysis.

We further investigated whether the immunomodulation of NKT cells with PBS57 could affect the recruitment of T cells into the diseased spinal cord before the onset of the disease. We therefore repeated the treatment regimen and found that 3 days after the third injection of PBS57, treated mSOD1 mice had significantly elevated proportions of T cells in the spinal cord, compared to their untreated mSOD1 littermates (**Fig. 6A**). There was also a slight increase in the proportion of NKT cells in the affected spinal cord following treatment, which was not statistically significant (**Fig. 6B**). Importantly, treatment with PBS57 did not affect the proportions of T cells in the spinal cord of WT animals, indicating that the mSOD1-associated pathology is a prerequisite for the recruitment of T cells into the spinal cord.

Collectively, these results suggest that there is a pathological activation of liver NKT cells in mSOD1 mice, which develops before the appearance of clinical symptoms of the disease, and precedes the accumulation of NKT cells in the spinal cord and lymphoid organs. In addition, modulation of NKT cells by PBS57 reverses the phenotype of NKT cells in the liver and results in an increased recruitment of T cells into the spinal cord, leading to a delay in the clinical onset of the disease.

## Discussion

In this study, we observed a robust increase in NKT cells in lymphoid organs and the spinal cord of mSOD1 mice along the pathway of disease progression. In addition, liver parenchyma underwent significant atrophy and lipid redistribution, together with marked changes in IGF-1 and its binding proteins. We further demonstrated that as a result of down-regulation of NKT-cell activity by PBS57, administered in a specific regimen, T cells were recruited into the spinal cord, disease onset was delayed, and lifespan was increased. Importantly, we tested only two PBS57 protocols, out of which only one showed a beneficial effect, though modest. Yet, these results suggest that further studies optimizing dose and timing may lead to a more effective treatment regimen.

For more than two decades, there has been evidence suggesting altered lipid metabolism and pathologic accumulation of gangliosides in the muscles and CNS of ALS patients [37], [38], [39]. In mSOD1 mice, increased lipolysis and depletion of adipose stores was reported, with a high-fat diet shown to prolong survival [40]. As such, hyperlipidemia was suggested as a positive prognostic factor predicting prolonged survival of ALS patients [41]. Accordingly, our finding that NKT cells are elevated in mSOD1 mice might stem from their activation by putative endogenous ligands in the periphery as a result of altered lipid metabolism. Alternatively, the increased proportion of NKT cells might result from lymphopenic conditions that progressively develop in ALS [10]. Moreover, the accumulation of NKT cells in the mSOD1 spinal cord might not necessarily depend on antigen specificity, as it was shown that the recruitment of NKT cells to CNS lesions in an animal model of multiple sclerosis could occur independently of CNS antigen presentation [42].

In addition to the marked neuroinflammatory component in the spinal cord in ALS, accumulating evidence suggests pathology of the peripheral immune system, as well [10], [13], [43], [44]. CD4^+^ T cells were shown to play a role in protecting neurons in general [8], [45], and MN in mSOD1 mice in particular [10], while immune deficiency in these mice resulted in reduced life expectancy [11], [12]. These findings are consistent with the concept of “protective autoimmunity”, developed by our group, stating that adaptive immunity is required to help control the detrimental local inflammatory response (involving mainly the resident microglia and possibly the scar forming astrocytes), which develops under acute or chronic neurodegenerative conditions in the CNS [5], [6], [7], [8], [46], [47]. Boosting of the protective autoimmune response with the random copolymer, glatiramer acetate, which weakly cross-reacts with self-antigens [48], was shown to have protective effect in animal models of chronic neurodegenerative diseases such as glaucoma and Alzheimer's disease when given infrequently (weekly or monthly but not daily) [5], [49], [50]. In the case of ALS, immunization with glatiramer acetate was shown to be beneficial only when emulsified in adjuvant in the low mSOD1 copy number model of ALS [51]. However, administration of glatiramer acetate has limited effects [10], [52], or even leads to adverse effects [53], when given without adjuvant or when administered repeatedly in the high mSOD1 copy number model of ALS, possibly as a result of immune suppression induced by this regimen [54].

Our present findings of elevated NKT cell levels in lymphoid organs of mSOD1 mice and an increase in the capacity of hepatic lymphocytes to secrete cytokines in response to NKT-cell stimulation, might indicate an additional aspect of the aberrant regulation of the immune response in ALS. Manipulating these cells by administration of PBS57 in the regimen used here resulted in a reduction in the number of NKT cells and their hyporesponsiveness, and concomitantly delayed appearance of clinical symptoms and prolonged survival. The approach undertaken in this study was aimed to demonstrate that NKT cells play an immunomodulatory role in a complex neurodegenerative disease such as ALS, therefore representing a novel therapeutic target for intervention. The apparent insufficiency of NKT cells in autoimmune diseases that are associated with an overwhelming adaptive immune response [15], [23], [25], [26], and their high numbers in ALS, a disease that is associated with compromised adaptive immunity [10], [13], suggest that this population of cells might be one of the non cell-autonomous factors in ALS [1], [2], [3], [33], [34], [35], [55]. To determine whether accumulation of these cells is a co-morbidity factor that causally contributes to disease progression, future studies involving the direct depletion of NKT cells must be conducted. Moreover, to establish whether NKT cell pathology is a general hallmark of ALS, these cells should be characterized in ALS patients, as well as in additional animal models of the disease, including a model of low copy number of mSOD1, and mice that overexpress the human WT SOD1 protein.

IGF-1 is a key neuronal survival protein, in general, and in ALS, in particular [29]. It is produced locally in the CNS by neurons and glia, but its main source in the circulation is the liver [56]. After liver injury, IGF-1 was shown to facilitate hepatocyte regeneration, and liver-specific IGF-1R knockout results in inhibition of this process [57]. The function of IGF-1 is tightly regulated by IGF-1 binding proteins, which can prolong its half-life in circulation (mainly IGFBP-3), but also inhibit its bioavailability [58]. Accumulating evidence suggest that IGFBPs have also intrinsic activities that are IGF-independent [59]. For instance, IGFBP-1 was shown to increase rapidly after partial hepatectomy, and was suggested to be a critical hepatic survival factor by reducing the levels of pro-apoptotic signals [60]. Thus the robust elevation of IGFBP-1 and the changes in IGF-1 axis observed in mSOD1 mice might reflect a local physiological attempt of hepatocyte regeneration in response to continuous liver damage. However, even if the increase in IGFBPs retards liver degeneration, it could limit the availability of circulating IGF-1 to MN. Interestingly, it was suggested that circulating IGF-1 can enter the CNS by means of low density lipoprotein receptor-related protein, megalin [61]. Our preliminary results indicate that mSOD1 mice had lower expression levels of megalin in the spinal cord during disease progression, as compared to WT controls (not shown). Importantly, at this stage we cannot determine if liver and IGF-1 related pathology contributes causally to the development of ALS symptoms, or if it is merely an epi-phenomenon reflecting systemic aberrations resulting from the ubiquitous expression of mSOD1 protein in this mouse model. Nevertheless, as there is also evidence for changes in the liver of patients with sporadic forms of MN disease (including ALS) [41], [62], these findings call for development of new therapies that will directly target the liver pathology, in addition to MN-based neuroprotection and systemic treatments aimed at reducing oxidative stress [63].

In addition to growth factors such as IGF-1, cytokines like TNF-α were also found to support liver regeneration [64]. However TNF-α secreted by activated NKT cells was shown to exacerbate liver injury [65]. Therefore, the overall increase in hepatic NKT cells might eventually result in liver inflammation and hepatocyte damage. Accordingly, we found that treatment with PBS57 attenuated liver atrophy and reduced the capacity of hepatic lymphocytes to secrete TNF-α, though it failed to reverse the reduction in hepatic IGF-1. This failure might reflect the apparently irreversible severe pathological changes in the liver, possibly due to mitochondrial damage similar to that occurring in MN [62], [66], [67], [68], [69]. Importantly, irreversible liver atrophy might explain the limited effect of the current treatment in mSOD1 mice as well as the limited success of many other potential ALS treatments tested. Nevertheless, it is still possible that elevation of NKT cells in the liver is part of a general attempt of the tissue to facilitate hepatocyte regeneration by means of TNF-α secretion.

Regardless of the reason as to why NKT cell levels and activation state increase in mSOD1 mice, their presence seemed to contribute to the failure to mount a protective T-cell response in the disease. NKT cells were shown to control effector T cells through various mechanisms including inhibition of IL-2 production, changing the properties of antigen presenting cells, and potentiation of Treg and myeloid suppressor cells [17], [24], [25], [70], [71], [72]. Administration of α-GalCer in the regimen used in the present study might result in an altered cytokine repertoire, together with anergy and even apoptosis of NKT cells, with possible lifting of T cell suppression [32], [36]. Interestingly, a high-fat diet, which was beneficial in ALS [40], was shown to induce apoptosis of NKT cells in the liver, via release of proinflammatory cytokines by immature myeloid cells [73]. Since repeated administration of glycolipid ligands may lead to NKT cell anergy and hyporesponsivness [32], it is possible that the therapeutic approach taken here converges into a common pathway of evoking a more efficient response of CD4^+^ T cells together with blocking the inflammatory response in the liver. In fact, we found that modulation of NKT cells by PBS57 induced recruitment of T cells into the spinal cord before the onset of clinical symptoms. In turn, such a T-cell response may lead to local production of IGF-1 in the spinal cord, by regulating microglial activity and recruitment of blood-born monocytes, collectively resulting in immunomodulation of the CNS milieu, and thereby favoring neuronal survival [5], [11], [12], [74], [75].

In conclusion, our understanding of the neuroinflammatory component in ALS is extended by the current observations that NKT cells are altered in the mSOD1 disease model. Evidence of liver inflammation and changes in IGF-1 axis together with the finding that modulating the activity of NKT cells is beneficial in mSOD1 mice, provide further support for the necessity of a proper immune response in ALS, and suggests novel secondary targets for therapeutic intervention in this disease and for other related neurodegenerative conditions.

## Supporting Information

Figure S1 **Peripheral changes in lymphoid populations in mSOD1 mice on C57Bl background.** FACS analysis of NKT and T cells from WT and mSOD1 mice at the progression (day 130) and end-stage (day 150) of the disease. The most prominent change was the increase in the proportion of NKT cells in all tissues tested, particularly in the liver. Data are expressed as mean±SEM (n = 7-12 for WT, n = 4-8 for mSOD1 at day 130, and n = 4-7 for mSOD1 mice at day 150).
(TIF)

Figure S2 **Alteration in lymphocyte distribution in the thymus and spleen of mSOD1 mice.** The proportion of hepatic iNKT cells was analyzed by FACS analysis of TCRβ expression, and binding of α-GalCer-loaded CD1d tetramers (CD1d/α-GC). Markers of activation (CD69), and migration (Ly6c) on gated T (R1) and NKT (R2) cells in the spleen and thymus of WT or mSOD1 mice at disease end-stage were tested by FACS. Both T and NKT cells from mSOD1 mice expressed higher levels of Ly6C as compared to WT, whereas CD69 was up-regulated only on NKT cells. Data are expressed as mean±SEM (n = 3-6 per group).
(TIF)

Figure S3 **PBS57 attenuates NKT cells elevation in the spleen and thymus of mSOD1 mice and boosts IFN-γ levels.** (**A**) The effect of PBS57 treatment was examined by monitoring the proportion of NKT, iNKT, and the activation state of NKT cells (defined by CD69 expression) in WT or mSOD1 mice at the end-stage of the disease by FACS. Values shown are normalized to those of WT controls. (**B**) IFN-γ levels in the spleen of WT or mSOD1 mice were determined by ELISA 3 days after the 3rd injection of PBS57. Data are expressed as mean±SEM (n = 3-6 per group). *p<0.05, **p<0.01 by Student's t-test, or one-way ANOVA followed by Student's t-test *post-hoc* analysis.
(TIF)
