# Supplementary figures and images for: Abnormal Changes in NKT Cells, the IGF-1 Axis, and Liver Pathology in an Animal Model of ALS
Source: PLoS One. 2011 Aug 2;6(8):e22374. doi: 10.1371/journal.pone.0022374 (PMC3149057; doi:10.1371/journal.pone.0022374)

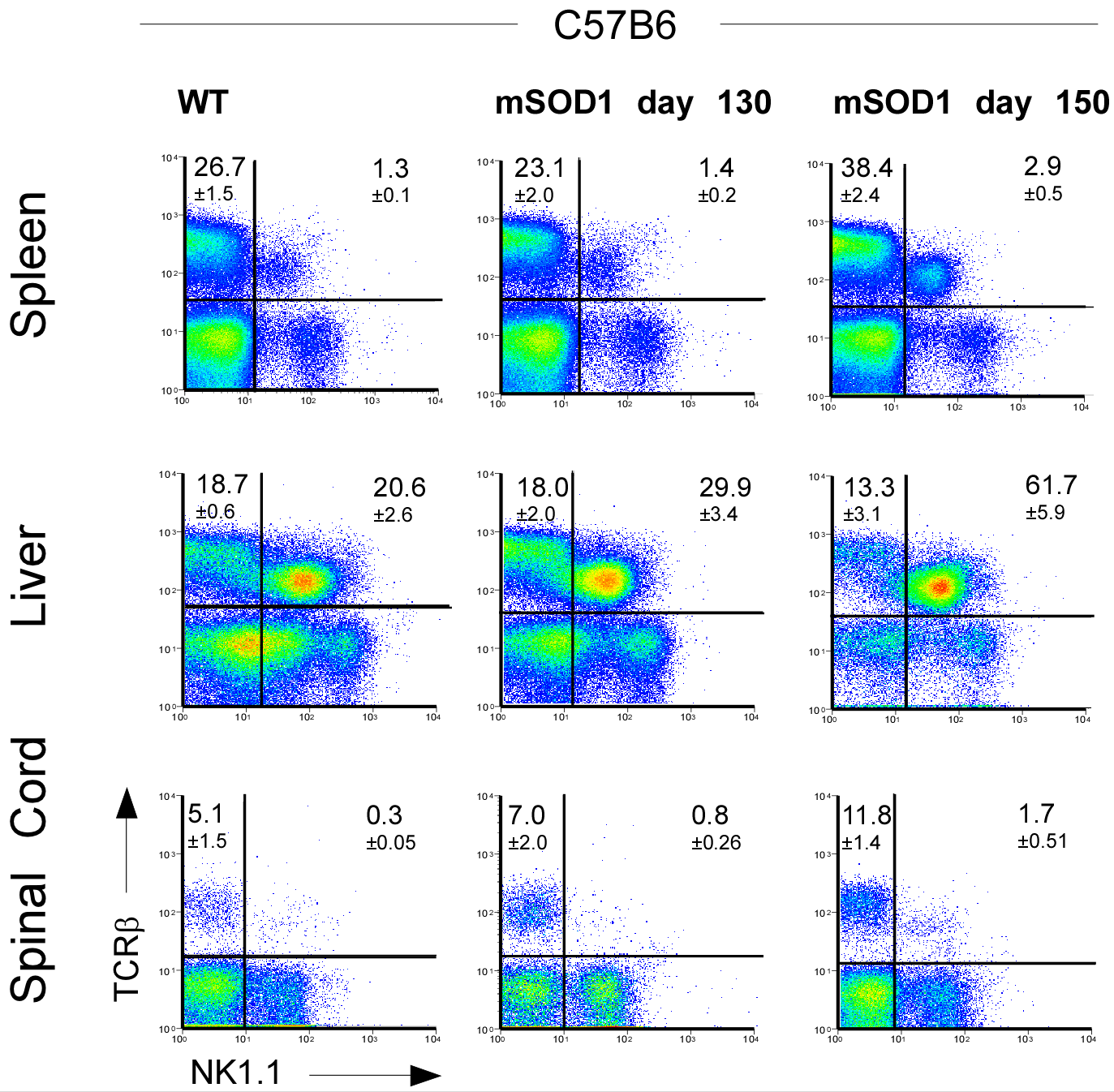

Supplement: Figure S1 — Peripheral changes in lymphoid populations in mSOD1 mice on C57Bl background. FACS analysis of NKT and T cells from WT and mSOD1 mice at the progression (day 130) and end-stage (day 150) of the disease. The most prominent change was the increase in the proportion of NKT cells in all tissues tested, particularly in the liver. Data are expressed as mean±SEM (n = 7-12 for WT, n = 4-8 for mSOD1 at day 130, and n = 4-7 for mSOD1 mice at day 150). (TIF) [file pone.0022374.s001.tif]

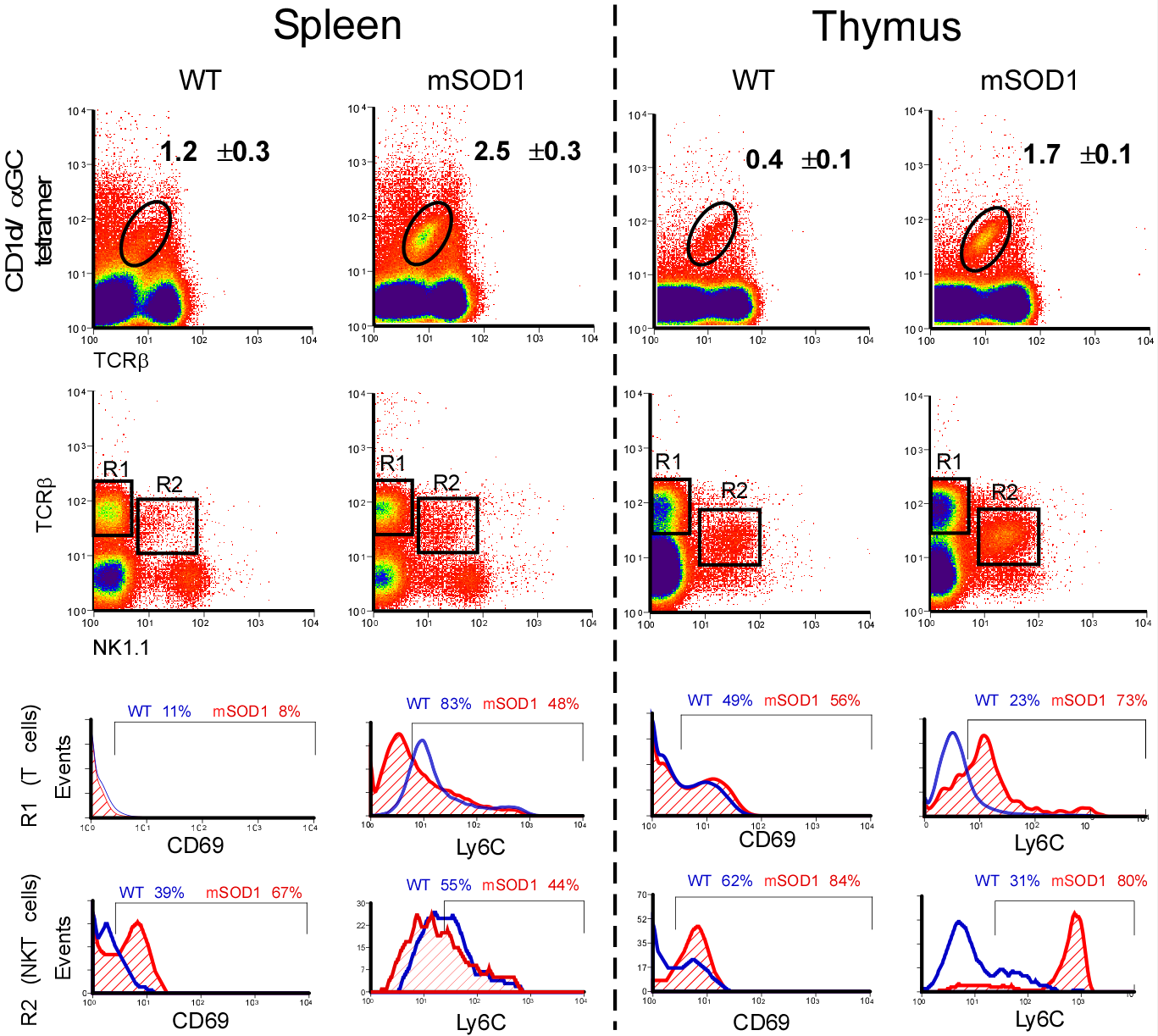

Supplement: Figure S2 — Alteration in lymphocyte distribution in the thymus and spleen of mSOD1 mice. The proportion of hepatic iNKT cells was analyzed by FACS analysis of TCRβ expression, and binding of α-GalCer-loaded CD1d tetramers (CD1d/α-GC). Markers of activation (CD69), and migration (Ly6c) on gated T (R1) and NKT (R2) cells in the spleen and thymus of WT or mSOD1 mice at disease end-stage were tested by FACS. Both T and NKT cells from mSOD1 mice expressed higher levels of Ly6C as compared to WT, whereas CD69 was up-regulated only on NKT cells. Data are expressed as mean±SEM (n = 3-6 per group). (TIF) [file pone.0022374.s002.tif]

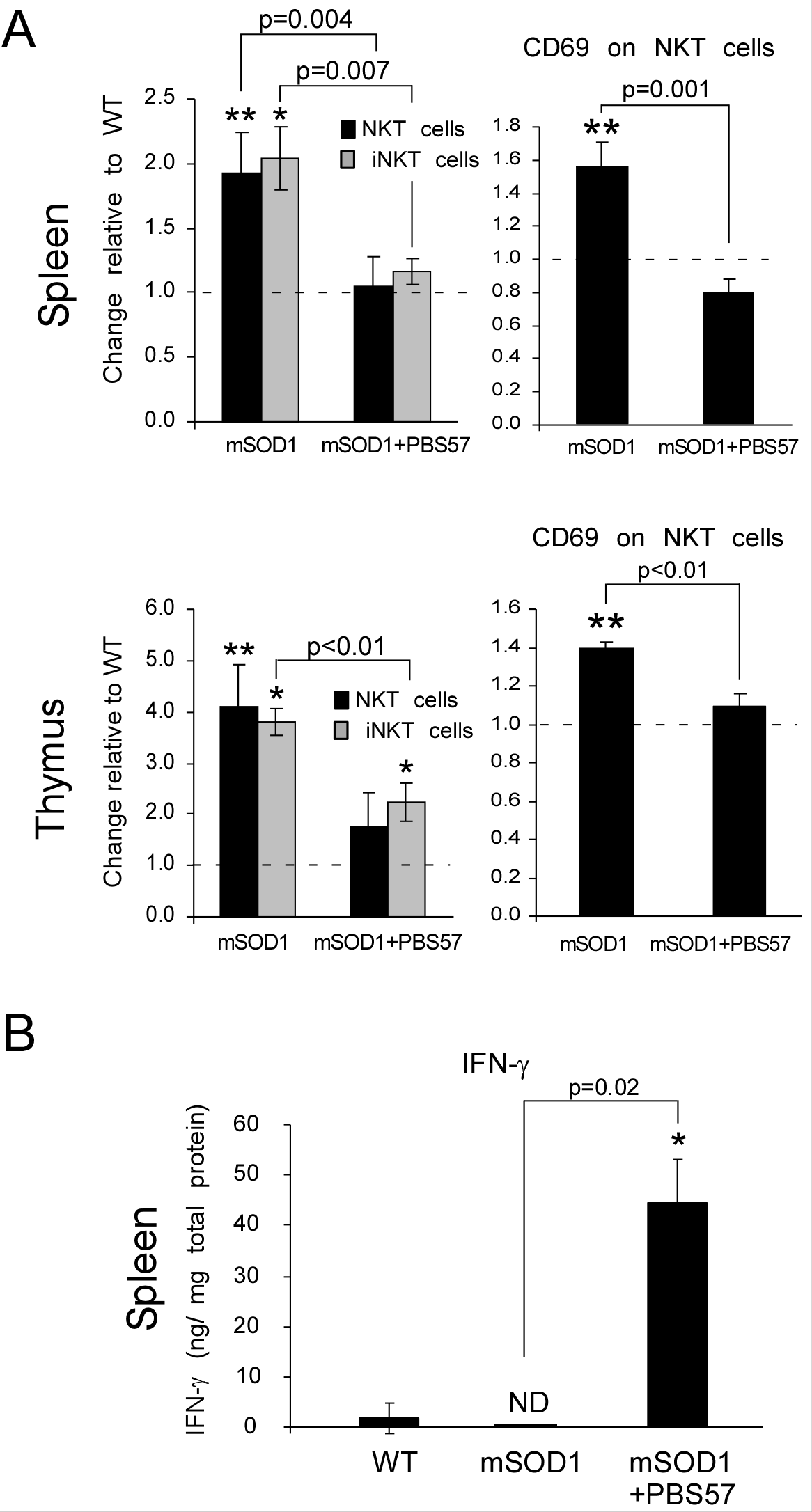

Supplement: Figure S3 — PBS57 attenuates NKT cells elevation in the spleen and thymus of mSOD1 mice and boosts IFN-γ levels. (A) The effect of PBS57 treatment was examined by monitoring the proportion of NKT, iNKT, and the activation state of NKT cells (defined by CD69 expression) in WT or mSOD1 mice at the end-stage of the disease by FACS. Values shown are normalized to those of WT controls. (B) IFN-γ levels in the spleen of WT or mSOD1 mice were determined by ELISA 3 days after the 3rd injection of PBS57. Data are expressed as mean±SEM (n = 3-6 per group). *p<0.05, **p<0.01 by Student's t-test, or one-way ANOVA followed by Student's t-test post-hoc analysis. (TIF) [file pone.0022374.s003.tif]
